# Investigating the Presence of Falsified and Poor-Quality Fixed-Dose Combination Artemether-Lumefantrine Pharmaceutical Dosage Forms in Kumasi, Ghana

**DOI:** 10.1155/2024/2650540

**Published:** 2024-03-25

**Authors:** Simon Nyarko, Kwabena Ofori-Kwakye, Raphael Johnson, Noble Kuntworbe, Denis Dekugmen Yar

**Affiliations:** ^1^Department of Pharmaceutics, Kwame Nkrumah University of Science and Technology, Kumasi, Ghana; ^2^Department of Public Health, Akenten Appiah-Menka University of Skills Training and Entrepreneurial Development, Mampong, Ghana

## Abstract

Artemether-lumefantrine (AL) is a highly effective and commonly used Artemisinin-based Combination Therapy (ACT) for treating uncomplicated malaria caused by *Plasmodium falciparum*, including drug-resistant strains. However, ineffective regulatory systems in resource-limited settings can lead to the infiltration of poor-quality and counterfeit antimalarial medicines into the pharmaceutical supply chain, causing treatment failures, prolonged illness, and disease progression. The objective of the study was to assess the quality of selected brands of fixed-dose combination (FDC) AL tablets and suspensions marketed in Kumasi, Ghana. A total of fourteen brands of FDC AL medicines, comprising eight tablets and six suspensions were purchased from various retail pharmacy outlets in Kumasi, Ghana. All samples were subjected to thorough visual inspection as a quick means of checking quality through meticulous observation of the packaging or dosage form. The quality parameters of the tablets were determined using uniformity of weight, hardness, friability, and disintegration tests. Suspensions were assessed based on pH and compared with the British Pharmacopeia (BP) standard. The samples were then analyzed for drug content (assay) using reverse-phase high-performance liquid chromatography (RP-HPLC). All the tablet samples conformed to BP specification limits for uniformity of weight (deviation of less than ± 5%), hardness (4.0–10 kg/mm^2^), friability (<1%), and disintegration time (<15 minutes). The active pharmaceutical ingredients' quantitative assay demonstrated that all the tablets met the BP specifications (90–110%). The results of the pH studies showed that out of the six brands of suspension investigated, five (83.3%) were compliant with the official specification for pH, while one (16.7%) failed the requirement. Unlike the tablet brands, drug content analysis of the six suspensions showed that two (33.3%) were substandard. The artemether and lumefantrine contents in these failed suspensions were variable (artemether: 81.31%–116.76%; lumefantrine: 80.35%–99.71%). The study results indicate that most of the tested products met the required quality standards, demonstrating satisfactory drug content and other quality specifications. The presence of substandard drugs underscores the necessity for robust pharmacovigilance and surveillance systems to eliminate counterfeit and substandard drugs from the Ghanaian market.

## 1. Introduction

Malaria is a life-threatening disease caused by a unicellular protozoan parasite that belongs to the genus *Plasmodium* [1]. Malaria has been a scourge on humans since antiquity and even now. Despite being preventable and treatable, malaria continues to have a devastating impact on people's health and livelihoods around the world [2]. Malaria is a major public health issue, particularly endemic to sub-Saharan Africa, where it is transmitted to humans via the bites of infected female *Anopheles* mosquitoes [3] and is a leading cause of illness and death in any cohort of humans [4]. In Africa, malaria is a major contributor to maternal and child mortality and the overall burden of disease. It is estimated that 90% of all malaria cases and 92% of all malaria deaths occur in Africa [5]. The disease asymmetrically affects children, expectant mothers, and people living in destitution [6].

In addition to its negative health effects, malaria has a greater economic impact on many endemic nations, which exacerbates the cycle of poverty and inhibits economic growth. It is estimated that direct losses from disease, medical care, and premature death cost Africa alone at least US$12 billion annually [7]. Malaria accounted for approximately 38% of outpatient visits, 27.3% of hospital admissions, and 48.5% of under-five deaths in Ghana in 2015, making it one of the leading causes of morbidity and mortality [8]. The availability, affordability, and quality of life-saving medications are still not guaranteed in low- and middle-income countries (LMICs), particularly in sub-Saharan Africa (SSA) [9], making the UN Sustainable Development Goal-3 (SDG-3) unsuccessful.

Pharmaceutical companies are playing an active role in the fight against malaria by producing ACTs following its approval and inclusion in the World Health Organization's Essential Medicines List (EML). This has led to the availability of several different brands of malaria drugs on the market [10]. Consumers are vulnerable to subpar or counterfeit antimalarials that are widely available on the market and even dispensed by chemists. It is estimated that up to 50% of the drugs circulating in some regions of Africa and Asia are counterfeit [11]. This issue undermines the efficacy of treatments for both chronic and infectious diseases and may lead to detrimental health consequences such as disease exacerbation, drug resistance, and mortality. Additionally, the use of poor-quality drugs may impose a significant financial burden on consumers, as it diverts resources towards ineffective or harmful therapies [12].

The World Health Organization (WHO) recommends AL as a first-line treatment for uncomplicated *P. falciparum* malaria in most endemic countries [13]. This is attributed to the high efficacy and favourable safety profile of AL. As a result, AL has become widely used and recognized as a valuable regimen in the global effort to combat malaria. The existence of multiple FDC AL tablets and suspensions has prompted concerns regarding potential variances in quality [14]. In the context of combating malaria and other diseases, the issue of poor-quality and falsified drugs presents a formidable challenge [15]. These drugs have the potential to be ineffective and even dangerous, undermining efforts to control and eliminate the disease [16]. Poor-quality drugs are those that fail to meet established standards for identity, purity, strength, and quality. They could have the incorrect active ingredient, too little or too much active ingredient, or be contaminated with other substances [17]. Counterfeit drugs are those with no active ingredient, the incorrect active ingredient, or drugs with the correct active ingredient but at the incorrect dosage. These medications may be ineffective in treating malaria and may even be harmful to patients. These drugs can be difficult to detect and can be found in various stages of the supply chain, from the manufacturer to the patient [18].

Several studies on substandard and falsified drugs have been conducted in some African countries, but only a few have been related to FDC AL tablets and suspensions [19]. One of the few studies on the quality of fixed-dose artemether and lumefantrine medicines in Ghana was conducted in Cape Coast by Prah and colleagues [20], who observed that physical examination of the package did not indicate counterfeiting, but 12.5% of the AL samples failed the HPLC assay test according to the International Pharmacopoeia (IP) [21] and this is a cause for concern of possible other similar drugs elsewhere in the country. Another study on children's essential medicines sampled from the Ashanti region of Ghana, which included artemether-lumefantrine fixed-dose suspensions, revealed that all (four) failed the content of the Active Pharmaceutical Ingredient (API) test and were substandard [22].

Regular monitoring of the quality of antimalarial medicines is critical in resource-limited settings to prevent the infiltration of poor-quality medicines into the pharmaceutical supply chain, which could jeopardize a positive treatment outcome [23]. The use of substandard or falsified drugs can have severe consequences for patient health and may lead to the development of drug resistance, treatment failure, and adverse reactions [24]. In low- and middle-income countries, the prevalence of poor-quality drugs in circulation is a significant concern, as patients may not have access to high-quality medications due to economic, social, or regulatory factors [12]. Therefore, the need for rigorous quality assessments of pharmaceutical products, including tablets and suspensions, cannot be overstated. This study, therefore, sought to assess the quality of different brands of antimalarial drugs containing fixed doses of artemether and lumefantrine marketed in Kumasi, Ghana. In this study examining postmarket drug quality parameters for tablets and suspensions, several key aspects were evaluated. For tablets, quality tests included disintegration time, hardness, friability, size, mass uniformity test, and API identification (drug assay). However, the study did not cover the dissolution test due to the failure of lumefantrine in most studies. Regarding suspensions, the official tests focused on pH and drug assay, while pourability and specific gravity were also assessed.

To combat the problem of poor-quality and falsified drugs, it is important to have robust regulatory systems in place to ensure that drugs meet established standards for quality and safety [25]. Quality assessment of FDC AL tablets and suspensions involves testing the products to ensure that they meet the established specifications for identity, purity, strength, and quality [23]. Assessing the quality of malaria drugs is critical to ensuring that patients have access to safe and effective treatments and to combating the global malaria burden. Quality assessment reveals issues such as poor content uniformity, which can lead to inconsistent dosing and reduced efficacy. Furthermore, quality assessment provides regulators and healthcare providers with the necessary information to make informed decisions about drug approval, procurement, and distribution [26]. Ultimately, the quality assessment of pharmaceutical products is vital in safeguarding public health and promoting global health equity.

## 2. Materials and Methods

### 2.1. Chemicals and Analytical Reference Standards

Primary analytical reference standards of purity ≥98% artemether and lumefantrine (HPLC grade, Sigma-Aldrich, USA), deionized water (gifted by Tradewinds Chemist Limited), analytical grade solvents including HPLC grade acetonitrile of purity ≥99.9% (LiChrosolv® Reag. Ph Eur. Supelco, Germany), and analytical grade orthophosphoric acid 85% (Supelco, Germany) were used. The drugs used in the study were purchased from various pharmacy outlet shops in Kumasi, Ghana. Artemether-lumefantrine brands coded LZT, MLT, LFT, and TMT were manufactured in Ghana, while LNT, IDT, and DMT were manufactured in India. Additionally, CTT tablets were manufactured in Turkey. The artemether-lumefantrine suspensions, coded LNS, IDS, BMS, and STS, were manufactured in India, while LFS and MLS were manufactured in Ghana.

### 2.2. Drug Sample Collection

A total of forty-one samples of fixed-dose combinations of AL, comprising fourteen different brands of medicines, including eight brands of oral tablets and six brands of oral suspensions, were analyzed in the study. The investigator selected five pharmacy outlets that were closer to markets and could serve a large populace. Out of these pharmacy outlets, two were selected from Tech Junction, one from Ayigya, one from New Tafo, and one from Kejetia. These sites were chosen for the study based on the size and availability of pharmacies, the economic activities of the area where these pharmacies were situated, and their accessibility to a greater number of people. The collection of drug samples took place from January 2023 to March 2023. All the brands included in the study had a remaining shelf life of at least 6 months, had been previously approved for marketing by relevant regulatory authorities, and were commercially available in the Kumasi metropolis at the time of sampling. The drug samples were collected in compliance with the Medicine Quality Assessment and Reporting guidelines (MEDQUARG) for reporting field surveys of medicine quality as proposed by Newton et al. to ensure robustness and transparency in the research process [27]. The guidelines stipulate that researchers must behave as typical consumers, thereby ensuring the study's purpose remains undisclosed. This includes purchasing medications in the same manner as any other shopper, rather than obtaining them under the guise of research. The MEDQUARG guidelines further mandate that packaging inspections are conducted and that the chosen analytical method is validated by recognized standards. The collected samples were properly labelled, stored, and transported to the laboratory for analysis, following appropriate protocols.

### 2.3. Visual Inspection of Drug Samples

The inspection criteria followed the guidelines outlined by Schiavetti and colleagues [28] and the WHO guidelines for pharmaceutical product packaging [29]. During the inspection, essential label information such as the dosage form, labelled strength, number of tablets per blister pack, country of manufacture, batch number, manufacturing date, expiry date, and Food and Drugs Authority (FDA) or product manufacturing license (PML) was carefully checked and recorded. The primary and secondary packaging conditions were examined for signs of tampering, damage, or deterioration. The researchers also analyzed the drug samples for attributes such as clarity, consistency, and the presence of particles, discoloration, or foreign matter. However, the study did not delve into security features like color-shifting inks, holograms, watermarks, DNA-based inks, taggants, or forensic analysis. Although not all physical properties were explicitly listed, Table 1 provides a summary of some observable features of the drug samples based on visual inspection.

### 2.4. Uniformity of Weight

The weight uniformity test of tablet samples was carried out following the established protocol outlined in the BP. A total of 20 tablets were randomly selected for each sample, and individual weighing was performed using a calibrated balance (Mettler Toledo, Model ML304T, Switzerland). The average weight of each sample was calculated by determining the sum of the weights of the 20 tablets and dividing it by 20. Additionally, the deviation of each tablet's weight from the average weight of the sample was calculated. The uniformity of mass for the uncoated tablet samples was assessed by comparing the obtained data against the specifications provided by the BP.

### 2.5. Determination and Quantification of the API Content by Reverse-Phase High-Performance Liquid Chromatography (RP-HPLC)

Six AL tablets were precisely weighed and their average mass was taken. The tablets were subsequently pulverized. An amount of powder equivalent to 30 mg of lumefantrine and 55 mg of artemether was weighed. The suspensions were reconstituted according to the manufacturer's guidelines. A volume containing an equivalent mass of 30 mg of lumefantrine and 55 mg of artemether was measured. The analytical solutions (for both tablet and suspension) were prepared by adding a precise volume of acetonitrile to the analyte, followed by sonication for 10 minutes. Subsequently, a 0.05% orthophosphoric acid solution was added to maintain a 70 : 30 v/v ratio of acetonitrile to orthophosphoric acid. The final mixture was subjected to additional sonication to enhance dissolution and was then filtered through 8 *μ*m pore size grade 2 qualitative filter paper (Whatman®). Before filtration, the vials used were thoroughly cleaned, rinsed, and dried [30].

A novel validated reverse-phase high-performance liquid chromatography method by Nyarko et al. in 2024 [30] was used to determine the percentage content of the APIs in fixed-dose combination tablet and suspension brands sampled. The HPLC analyses were carried out on an Agilent 1260 Infinity Series HPLC system (Agilent Technologies, Santa Clara, California, USA), equipped with a quaternary pump, autosampler, variable wavelength detector (VWD), and an ODS Intersil-C8 column (Phenomenex) (150 × 4.6 mm, 5.0 mm particles). The HPLC was controlled by a PC workstation (HP) using ChemStation software. The column temperature, flow rate, injection volume, and run-time were 25°C, 1.0 ml/min, 5.0 *μ*l, and 6 min, respectively. Column temperature was maintained at 25°C. UV detection was performed at 210 nm.

### 2.6. Size (Thickness and Diameter Uniformity) of Tablets

The thickness and diameter of tablet samples were determined according to the method given in the BP [31]. Randomly selected tablets (*n* = 10) from each sample were individually measured with a calibrated Vernier caliper (Mitutoyo, Absolute Digimatic, Model CD-8″ CSX, Japan). The reading is automatically displayed on the screen of the device.

### 2.7. Disintegration Time Test

The disintegration time test of six randomly selected tablets was determined in about 900 mL distilled water at 37 ± 2°C using Tab-Machines (Mumbai, India) disintegration test apparatus in line with the specification stated in the BP.

### 2.8. Hardness (Breaking Strength)

Ten (10) tablets were randomly selected from each brand and the breaking strength of each tablet was determined with a VEEGO hardness tester (Alson Tech Services, India). The mean ± SD value of each sample was determined. The hardness test of the uncoated tablet samples was assessed by comparing the obtained data against the specifications in the BP.

### 2.9. Friability Test

The friability (%) of randomly selected tablets for each sample brand was determined using Campbell Electronics (Mumbai, India) friabilator operated at 25 revolutions per minute (rpm) for 4 minutes, thus 100 revolutions. A sample size of tablets, totaling 6.5 grams in weight, was weighed for testing. The friability tests of the uncoated tablet samples were assessed by comparing the obtained data against the specifications provided in the BP.

### 2.10. Determination of pH after Reconstitution of Suspensions

The pH measurement of each sample was conducted using a calibrated Hanna pH meter (model HI 2215, Hanna Instruments, UK) after reconstitution as per the manufacturer's instructions. The pH reading was taken 15 minutes after reconstitution, and the obtained values were subsequently compared with the pH specifications outlined in the British Pharmacopoeia for each respective sample.

### 2.11. Determination of the Ease of Pourability of Suspensions

The pourability of suspensions was assessed to determine their ease of pouring. This evaluation involved observing the flow behavior of the suspensions when poured from their containers. The container of suspensions was held at a suitable angle and poured into a beaker. Observations regarding the flow characteristics, such as smoothness, resistance, or clumping, were made. Scores on a scale from 1 to 4 were given to the samples during pouring: very easy pourability (4), easy pourability (3), moderate pourability (2), and difficult pourability (1).

### 2.12. Determination of Specific Gravity of Suspensions

The specific gravity of each suspension sample was determined with a 50 mL pycnometer. The mass of the empty pycnometer and the combined mass of the pycnometer and deionized water were determined. The specific gravity of each sample was then computed by comparing the weight of the drug to the weight of deionized water.

### 2.13. Data Analysis and Reporting

GraphPad Prism for Windows version 8 (GraphPad Software, San Diego, CA, USA) and Microsoft Excel 2019 were used for all statistical analyses. Data obtained are expressed as mean ± SEM, RSD, and percentages. For sampling, MEDQUARG guidelines for reporting field surveys of the quality of medicines as proposed by Newton et al. [27] with minor modifications were utilized.

## 3. Results

### 3.1. Visual Inspection of Drug Samples

Following rigorous research methodologies, a thorough visual examination of both the packaging and the drug products was conducted after their procurement from various pharmacy outlets. The study encompassed a total of forty-one drug products, comprising twenty-nine (70.7%) AL oral tablets and twelve (29.3%) AL oral suspensions, which were further categorized into fourteen distinct brands, serving as samples for analysis. Among the samples, eight (8) tablet brands were identified, with four (50%) being locally manufactured and four (50%) foreign manufactured. Additionally, six suspension brands were included, consisting of four (66.7%) foreign-manufactured and two (33.3%) locally manufactured suspensions. Thirteen (93%) of the test samples conformed to the visual inspection criteria stipulated by the WHO guidelines, except sample MLT which had no product license number and FDA number on the packaging. The shelf life of the samples ranged from 2 to 3 years. The data on visual inspection are displayed in Table 1.

### 3.2. Uniformity of Weight of Tablets

The weight variation test of the AL tablet samples revealed that the percentage weight deviation of the tablets from their respective mean weights was less than 5% (Table 2). This complies with the BP specification, i.e., the deviation of the individual tablet masses should not exceed ±10% for tablets with an average weight of ≤80 mg, ±7.5% for tablets weighing between ≥80 mg and ≤250 mg, and ±5% for tablets weighing ≥250 mg [31].

### 3.3. Analysis of Artemether-Lumefantrine Market Samples (Quantitative Estimation of API Content)

Assay analysis was performed on the sampled marketed drug products to investigate the content and concentration of the API present in them. The quantity of the two APIs was estimated using the calibration curve equation obtained from the HPLC analysis.  Table 3 presents the results of the analyzed market samples using the developed RP-HPLC method.

### 3.4. Thickness and Diameter Uniformity of Tablets

The diameter of the tablets ranges from 12.05 ± 0.07 mm to 21.13 ± 0.11 mm with a deviation of less than ±5% (Table 4). This conforms to the BP specification (deviation of an individual unit from the mean diameter should not exceed ±5% for a diameter of less than 12.5 mm and ±3% for a diameter of 12.5 mm or more). Similarly, the thickness ranges from 5.67 ± 0.10 mm to 6.85 ± 0.10 mm with a deviation of less than ±5% for all samples.

### 3.5. Hardness, Friability, and Disintegration Time

The hardness, friability, and disintegration time of the samples were assessed and analyzed following the BP guidelines (Table 4). The obtained data for hardness exhibited a range of 4.34 ± 1.23 kg/mm^2^ to 8.40 ± 0.67 kg/mm^2^. The friability of the samples was found to be less than 1%, indicating good structural integrity. Furthermore, the disintegration time for all samples was observed to be less than 15 minutes, with a range of 2 minutes to 13 minutes. These results align with the criteria outlined in the BP, which specifies a hardness range of 4.0 kg/mm^2^ to 10.0 kg/mm^2^, a maximum friability of 1%, and a disintegration time of less than 15 minutes for uncoated tablets. Therefore, based on the analysis of these parameters, the samples meet the specified requirements.

### 3.6. pH, Pourability, and Specific Gravity of the Suspensions

The pH values of the reconstituted samples exhibited a range of 4.81 ± 0.21 to 7.39 ± 0.02. Notably, out of the six suspension samples evaluated, only one sample failed to meet the acceptable range (4.5–6.8) for maximum stability of fixed-dose AL suspensions. To further assess the pourability of the reconstituted suspensions, a scale ranging from 1 to 4 was employed. Based on this scale, the distribution of pourability ratings was as follows: two (33.3%) of the samples were classified as having very easy pourability (rating of 4), three (50%) were classified as having easy pourability (rating of 3), and the remaining sample was deemed to have moderate pourability (rating of 2). Importantly, none of the evaluated samples demonstrated difficult pourability. The specific gravity values varied from 1.07 to 1.14 and may provide insight into the absence of pourability issues observed across all the samples. These results are shown in Table 5.

## 4. Discussion

The packaging of a medication plays a crucial role in preserving its quality and the integrity of the Active Pharmaceutical Ingredient (API) contained within. A detailed inspection of the physical attributes of a medicine's packaging is vital for assessing the overall quality of the drug [32]. Apart from a package providing containment and protection for drugs, it must also provide information that is useful to patients and clinicians [33]. The type of packaging material, labels, presence or absence of anti-counterfeit features, and information leaflets are crucial in assessing the quality of a product. Key information such as the manufacturing date, expiry date, storage condition, ingredients list, manufacturer address, and indications must be included in a good packaging system [34]. This study revealed that the shelf life across all brands ranged from 2 to 3 years which differs a bit from a study by Prah et. [35] who in their study found the shelf life ranging from 2 to 4 years. Similar to an observation made by Prah et al., [36] and Belew et al., [23], all drugs were registered for use in Ghana. None of the brands showed an advanced form of anti-counterfeit feature such as forensic techniques. The common anti-counterfeit techniques observed were overt or semiovert which include on-product marking, holograms, and security graphics. The inability of manufacturers to include advanced covert features in their products could be because of the high cost that would be involved in adopting such advanced technologies [36].

The quality of tablets plays a critical role in their therapeutic efficacy [37]. Good quality features of drugs assure high therapeutic outcomes in patients [38]. Regulatory agencies establish guidelines to ensure that pharmaceutical companies meet the regulatory specifications for the production of quality, safe, and effective medicines. In this current research, the weight variation test of the samples studied revealed that the percentage weight deviation of all the brands was less than 5%. This complies with the specification set by the BP [31] and all the tablets passed the weight uniformity test. However, this is not in agreement with the observation made in Uganda by Ocan et al. [39] who found that 1/74 (1.4%) failed the weight uniformity test. It is recommended that the weight deviation among individual tablets of the same batch should not exceed the specification stated by the various Pharmacopeia (BP: NMT 5% for tablets whose weight is ≥250 mg; USP: NMT 5% for tablets whose weight is ≥324 mg). There is a correlation between weight and content uniformity. The better the weight uniformity is, the higher the likelihood that the API(s) is/are uniformly distributed in the medicine [40]. Interestingly, the findings of this research revealed that weight uniformity is a crucial factor in pharmaceutical quality control and has a significant influence on the dosage accuracy, efficacy, and safety of the drug. This relationship is crucial because significant variations in tablet weight could suggest an uneven distribution of the API. Such inconsistencies could lead to some tablets containing more or less of the active ingredient than intended, potentially resulting in subtherapeutic or toxic effects [41, 42]. Weight uniformity ensures that each unit of a drug product consistently contains the intended amount of the API. In this study, all tablets passed the weight uniformity test and met the BP specification of 90%–110% for drug content. This finding suggests a high level of quality control in the manufacturing process. It indicates that the API is evenly distributed throughout each tablet, ensuring consistent dosing and potentially enhancing therapeutic efficacy. This consistency in weight and drug content uniformity is a positive indicator of the quality of the tablets. It suggests that the manufacturing process is robust and capable of producing reliable and safe medication for patients.

On the other hand, it was observed that two out of the six suspension brands did not pass the content uniformity test. Suspensions are alternatives for children and adults who possibly have issues with swallowing. Failure to meet content uniformity specifications could lead to variations in drug efficacy and safety. The failure of these two brands to meet the content uniformity standards suggests potential issues in the manufacturing process. It could be due to factors such as inadequate mixing, improper formulation, excipient incompatibility, or issues with the manufacturing equipment. These inconsistencies could lead to variations in the concentration of the API in different batches or even within the same batch.

The diameter and thickness of the tablets were within the acceptable deviation of ±5%. The hardness ranged from 4.34 ± 1.23 kg/mm^2^ to 8.40 ± 0.67 kg/mm^2^, aligning with the BP's specified range of 4.0 kg/mm^2^ to 10.0 kg/mm^2^. The friability was less than the maximum allowable 1%, and the disintegration time was under the required 15 minutes (2 ± 0.03–13 ± 0.13). These results indicate that all samples met the BP criteria, suggesting a positive impact on the bioavailability of the active pharmaceutical ingredient due to the influence of the disintegration rate on dissolution and absorption [43]. The observation agrees with similar research conducted by Prah et al. [20] in Cape Coast, Ghana. Their research found that the percentage weight deviation of eight brands of FDC AL tablets from their respective mean weight was less than 10%. Also, according to their findings, the tablets' disintegration in an aqueous medium was in the range of 9 ± 0.3–14 ± 0.8 minutes (<15 minutes) and the breaking strength ranged from 3.4 kPa to 5.3 kPa and the percentage friability was less than 1% (0.01–0.23% w/w). Thickness and diameter are two crucial parameters that are used in studying tablet quality. They play a role in the volume and surface area of tablets which in turn has a relation with the amount of API present and the strength and integrity of other ingredients in tablets [44]. It is worth mentioning that the size of tablets has an impact on ease of swallowing, especially in the case of children [45]. To ensure patient compliance, manufacturers should factor in minimizing the thickness and diameter of tablets when designing dosage forms. Poor disintegration time means the drug cannot be released from a tablet matrix or granules and becomes available for dissolution. Also, excessive friability can indicate weak or poorly manufactured tablets. Appropriate friability and hardness imply that the tablets can withstand mechanical stress during handling, transportation, and packaging [46].

The pH values of the reconstituted AL suspensions ranged from 4.81 ± 0.21 to 7.39 ± 0.02. Interestingly, one out of the six evaluated suspension samples did not meet the BP specification limit for pH. This finding contradicts the study conducted in [22], which found that three out of four (75%) brands of FDC AL essential children's medicine failed the pH specification test, while only one (25%) passed. The pH level can influence the stability, compatibility, and therapeutic effectiveness of suspensions. Some excipient particles may be pH-sensitive, leading to aggregation, precipitation, or degradation of the suspension if the pH is not within the optimal range, resulting in poor therapeutic effectiveness.

The pourability of the reconstituted suspensions was assessed using a scale from 1 to 4. According to this scale, two (33.3%) of the samples were classified as having very easy pourability (rating of 4), three (50%) were classified as having easy pourability (rating of 3), and the remaining sample was deemed to have moderate pourability (rating of 2). Notably, none of the evaluated samples demonstrated difficult pourability. Pourability measures the suspension's ability to flow when poured out of its container. Poor pourability can lead to inaccurate dosing due to inconsistent volume or a difficult-to-pour suspension. A suspension with good pourability facilitates accurate measurement and dosage administration, especially for children or elderly patients who may struggle with viscous or poorly flowing suspensions [47]. The specific gravity values of the suspensions, which varied from 1.07 to 1.14, may shed light on the absence of pourability issues observed across all the samples. The specific gravity of a substance measures its density relative to water, with values greater than 1 indicating a denser material. The specific gravity values obtained in this study suggest that the samples had varying degrees of density, but none were excessively heavy or light. The specific gravity of a suspension can influence its stability by affecting particle settling or sedimentation. If the specific gravity of the particles and the suspending medium are similar, it can help prevent settling or maintain a uniform distribution of the API throughout the suspension [48].

## 5. Conclusion and Recommendations

The study results indicate that a greater proportion of the tested products meet the required quality standards, demonstrating satisfactory drug content and other quality characteristics. However, it is concerning that some of the foreign-manufactured suspensions did not meet the standards for drug content and pH. The study indicates that substandard artemether-lumefantrine drugs may be entering Ghana undetected, raising concerns about their effectiveness. It underscores the need for regulatory authorities to enforce strict quality control measures and Good Manufacturing Practices (GMPs). The findings provide valuable insights for healthcare professionals and consumers, aiding informed decisions about drug selection and use. This contributes to the fight against malaria and improves public health. Future research should broaden its scope beyond Kumasi to ascertain a national prevalence of poor-quality or counterfeit fixed-dose combination artemether-lumefantrine dosage forms across the country.

## Figures and Tables

**Table 1:** Visual inspection of packaging of artemether-lumefantrine fixed-dose combination medicines.

Sample code and dosage form	Labelled strength	No. of tablets per blister pack	Country of manufacture	Batch No.	Mfg. date	Exp. date	FDA/PML number
LZT							
Tablet 1	20 mg/120 mg	24	Ghana	HIXO03	03/2022	03/2024	Yes
Tablet 2	20 mg/120 mg	24	Ghana	HIXO03	03/2022	03/2024	Yes
LNT							
Tablet 3	80 mg/480 mg	6	India	B1AFM016	02/2022	01/2025	Yes
Tablet 4	80 mg/480 mg	6	India	B1AFM016	02/2022	01/2025	Yes
Tablet 5	80 mg/480 mg	6	India	B1AFM016	02/2022	01/2025	Yes
Tablet 6	80 mg/480 mg	6	India	B1AFM030	04/2022	03/2025	Yes
Tablet 7	80 mg/480 mg	6	India	B1AFM030	04/2022	03/2025	Yes
Tablet 8	20 mg/120 mg	24	India	A1AFJO94	07/2021	06/2024	Yes
MLT							
Tablet 9	80 mg/480 mg	6	Ghana	1711Y	11/2022	11/2024	No
Tablet 10	80 mg/480 mg	6	Ghana	1711Y	11/2022	11/2024	No
Tablet 11	80 mg/480 mg	6	Ghana	1711Y	11/2022	11/2024	No
Tablet 12	80 mg/480 mg	6	Ghana	1711Y	11/2022	11/2024	No
Tablet 13	80 mg/480 mg	12	Ghana	0503Y	03/2022	03/2024	No
LFT							
Tablet 14	80 mg/480 mg	6	Ghana	T017F024	04/2022	03/2025	Yes
Tablet 15	80 mg/480 mg	6	Ghana	T017F024	04/2022	03/2025	Yes
Tablet 16	80 mg/480 mg	6	Ghana	T017F019	04/2022	03/2025	Yes
Tablet 17	80 mg/480 mg	6	Ghana	T017F050	12/2022	11/2025	Yes
Tablet 18	80 mg/480 mg	6	Ghana	T017F050	12/2022	11/2025	Yes
CTT							
Tablet 19	80 mg/480 mg	6	Turkey	KCU43	07/2021	06/2023	Yes
Tablet 20	80 mg/480 mg	6	Turkey	KDE81	09/2021	08/2023	Yes
Tablet 21	80 mg/480 mg	6	Turkey	KDE81	09/2021	08/2023	Yes
Tablet 22	80 mg/480 mg	6	Turkey	KDE81	09/2021	08/2023	Yes
TMT							
Tablet 23	80 mg/480 mg	6	Ghana	BN001	07/2021	07/2025	Yes
Tablet 24	80 mg/480 mg	6	Ghana	BN001	07/2021	07/2025	Yes
Tablet 25	80 mg/480 mg	6	Ghana	BN001	07/2021	07/2025	Yes
IDT							
Tablet 26	80 mg/480 mg	6	India	I-56001	01/2022	12/2024	Yes
Tablet 27	80 mg/480 mg	6	India	I-56001	01/2022	12/2024	Yes
DMT							
Tablet 28	80 mg/480 mg	6	India	LU22007	02/2022	01/2025	Yes
Tablet 29	80 mg/480 mg	6	India	LU22007	02/2022	01/2025	Yes
LNS							
Suspension-1	240 mg/1440 mg/5 ml	N/A	India	B1AFM020	03/2022	03/2024	Yes
Suspension-2	240 mg/1440 mg/5 ml	N/A	India	B1AFM020	03/2022	03/2024	Yes
MLS							
Suspension 3	20 mg/120 mg/5 ml	N/A	Ghana	0701Z	01/2023	12/2025	Yes
Suspension 4	20 mg/120 mg/5 ml	N/A	Ghana	7308Y	08/2022	08/2024	Yes
LFS							
Suspension 5	20 mg/120 mg/5 ml	N/A	Ghana	D003F094	09/2022	09/2025	Yes
Suspension 6	20 mg/120 mg/5 ml	N/A	Ghana	D003F094	09/2022	09/2025	Yes
IDS							
Suspension 7	20 mg/120 mg/5 ml	N/A	India	I-57001	01/2021	12/2023	Yes
Suspension 8	20 mg/120 mg/5 ml	N/A	India	I-57001	01/2021	12/2023	Yes
BMS							
Suspension 9	20 mg/120 mg/5 ml	N/A	India	PDS01-01	01/2023	01/2015	Yes
Suspension 10	20 mg/120 mg/5 ml	N/A	India	CDS09-02	01/2021	12/2023	Yes
STS							
Suspension 11	20 mg/120 mg/7 ml	N/A	India	1390423	06/2021	06/2024	Yes
Suspension 12	20 mg/120 mg/7 ml	N/A	India	1390423	06/2021	06/2024	Yes

N/A = not applicable. FDA = Food and Drugs Authority, Ghana. PML = product manufacturing license. Mfg. date = manufacturing date. Exp. date = expiry date.

**Table 2:** Weight variation test.

Samples	Weight of 20 tablets (g (*x*))	Average weight (g (*x*/20))	^*∗*^Standard deviation (±)	% RSD	Inference
LZT	7.3834	0.3692	0.0177	4.794	Passed
LNT	13.7309	0.6865	0.0078	1.132	Passed
MLT	19.8272	0.9914	0.0166	1.674	Passed
LFT	13.4692	0.6735	0.0117	1.737	Passed
CTT	19.3590	0.9680	0.0040	0.413	Passed
TMT	17.0451	0.8523	0.0097	1.138	Passed
IDT	22.7536	1.1377	0.0163	1.433	Passed
DMT	14.7696	0.7385	0.0073	0.948	Passed

% RSD = percentage relative standard deviation. ^*∗*^Acceptance criteria (BP): NMT ± 5%.

**Table 3:** Results of analyses of market samples using the developed RP-HPLC method.

Formulation	Sample code	Retention time L/A (min)	^*∗*^Percentage content of artemether (%) ± SD	^Ψ^Percentage content of lumefantrine (%)	Remarks on API content
Tablet	LZT	1.44/5.38	97.76 ± 1.03	91.81 ± 1.14	Passed
	LNT	1.41/5.38	99.68 ± 0.89	97.55 ± 1.05	Passed
	MLT	1.39/5.36	99.50 ± 0.23	98.33 ± 1.23	Passed
	LFT	1.40/5.38	99.49 ± 0.18	96.83 ± 0.28	Passed
	CTT	1.41/5.37	99.89 ± 0.76	99.12 ± 0.97	Passed
	TMT	1.39/5.38	96.90 ± 0.25	91.64 ± 1.11	Passed
	IDT	1.40/5.38	94.94 ± 0.06	92.16 ± 1.26	Passed
	DMT	1.39/5.38	99.68 ± 0.34	96.52 ± 0.73	Passed

Suspension	MLS	1.42/5.30	98.37 ± 0.54	99.71 ± 0.27	Passed
	LFS	1.42/5.40	99.92 ± 0.32	97.98 ± 1.09	Passed
	IDS	1.42/5.40	100.10 ± 0.25	91.27 ± 1.16	Passed
	LNS	1.42/5.43	98.50 ± 0.88	97.72 ± 1.02	Passed
	STS	1.42/5.42	116.76 ± 0.94	80.35 ± 1.01	Failed
	BMS	1.42/5.43	88.31 ± 0.44	82.08 ± 0.98	Failed

*L* = lumefantrine. *A* = artemether. ^*∗*^Acceptance criteria (BP): 90%–110%. ^Ψ^Acceptance criteria (BP): 90%–110%.

**Table 4:** Quality tests for fixed-dose AL tablets.

Samples	Diameter (mm) ± SD	Thickness (mm) ± SD	^*∗*^Hardness (kg/mm^2^) ± SD	^Ψ^Friability (%)	^¥^Disintegration time (min) ± SD
LZT	16.49 ± 0.33	5.67 ± 0.10	5.43 ± 1.11	0.11	13 ± 0.13
LNT	12.05 ± 0.09	5.94 ± 0.09	7.00 ± 0.85	0.01	5 ± 0.24
MLT	19.18 ± 0.07	6.85 ± 0.10	8.52 ± 0.52	0.02	4 ± 0.05
LFT	12.57 ± 0.60	5.81 ± 0.11	4.34 ± 1.23	0.66	2 ± 0.11
CTT	20.24 ± 0.04	5.86 ± 0.08	8.40 ± 0.67	0.05	2 ± 0.03
TMT	18.46 ± 0.06	5.81 ± 0.11	5.62 ± 0.58	0.15	5 ± 0.18
IDT	21.13 ± 0.11	5.68 ± 0.09	7.58 ± 0.95	0.18	12 ± 0.01
DMT	16.49 ± 0.33	5.67 ± 0.10	6.92 ± 0.57	0.50	3 ± 0.16

^*∗*^Acceptance criteria (BP) = 4–10 kg/mm^2^. ^Ψ^Acceptance criteria (BP) = NMT 1%. ^¥^Acceptance criteria (BP) = NMT 15 minutes.

**Table 5:** Quality tests for fixed-dose AL suspensions.

Samples	^*∗*^pH ± SD	Remarks on pH	Specific gravity	^ϯ^Ease of pourability (1–4)
MLS	4.81 ± 0.21	Passed	1.08	4
LFS	5.07 ± 0.14	Passed	1.07	4
IDS	7.39 ± 0.02	Failed	1.11	3
LNS	5.04 ± 0.18	Passed	1.10	3
STS	5.87 ± 0.04	Passed	1.14	2
BMS	5.56 ± 0.10	Passed	1.12	3

^*∗*^Acceptance criteria (BP): artemether-lumefantrine (4.5–6.8). ^ϯ^Rating on a scale of 1–4 (4 = very easy pourability, 3 = easy pourability, 2 = moderate pourability, and 1 = difficult pourability).

## Data Availability

The data underlying the findings of this study are available within the article. Raw data that support the findings of this study are available from the authors, upon written request.
